# A convenient allylsilane-*N*-acyliminium route toward indolizidine and quinolizidine alkaloids

**DOI:** 10.1186/1860-5397-3-32

**Published:** 2007-10-02

**Authors:** Roland Remuson

**Affiliations:** 1 UMR 6504, CNRS Université Blaise Pascal (Clermont-Fd), 63177 Aubière Cédex, France

## Abstract

This review relates all the results that we obtained in the field of the total synthesis of indolizidine and quinolizidine alkaloids using a strategy of the addition of an allylsilane on an *N-*acyliminium ion. In this paper, we describe the synthesis of racemic indolizidine 167B and chiral indolizidines: (-)-indolizidines 167B, 195B, 223AB, (+)-monomorine, (-)-(3*R*,5*S*,8a*S*)-3-butyl-5-propylindolizidine and (-)-dendroprimine. Next, we relate the synthesis that we have developed in the quinolizidines field: (±)-myrtine and epimyrtine, (±)-lasubines I and II and chiral quinolizidines: (+)-myrtine, (-)-epimyrtine, (-)-lasubines I and II and (+)-subcosine II.

## Background

Bicyclic indolizidines and quinolizidines are commonly occurring structural skeleta found in natural alkaloids. Such compounds have been isolated from animals: poison frogs of the family *Dendrobatidae* have provided a rich source of novel pharmacologically active alkaloids, including a variety of bicyclic nitrogen heterocyclic compounds such as indolizidines. [1–2] Several quinolizidine alkaloids have been isolated from plants: *Lythraceae* family (Lasubines), [3]*Vaccinum myrtillus* (myrtine, epimyrtine). [4–5]

Firstly, most of these compounds are frequently found in concentrations too low to allow complete structural elucidation; secondly, the biological activities for most of them make these alkaloids ideal targets for total synthesis.

**Scheme 1:**
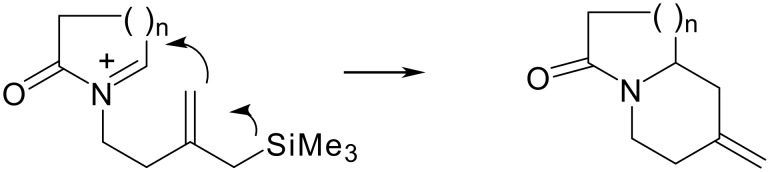
Allylsilane-*N*-acyliminium cyclisation.

We have developed a new method to generate bicyclic indolizidine and quinolizidine compounds based on an intramolecular cyclisation of acyliminium ions substituted by an allylsilyl side chain as an internal π-nucleophile (Scheme 1). [6]

This reaction has proven to be a very powerful method for construction of indolizidine and quinolizidine ring systems with efficient control of stereochemistry.

## I Indolizidines

### I 1. Indolizidine 167B

Indolizidine 167B, one of the simplest amphibian indolizidine alkaloids, was originally found as a trace component in the skin secretions of a frog belonging to the genus Dendrobates captured on the Isla Colon Panama. [7] The structure and relative stereochemistry shown in **1** are now accepted as correct although the absolute configuration of the natural product remains uncertain. [8] The lack of availability of the natural material and the important biological activities of the compound make this alkaloid an ideal target for total synthesis. [9–16]

#### I 1.1 Intramolecular cyclisation

We have found that intramolecular cyclisation of an allylsilane on an acyliminium ion constituted an excellent route to nitrogen bicyclic ring systems. [6] This method represents an efficient and stereoselective strategy for the preparation of 5-substituted indolizidines.

**Scheme 2:**
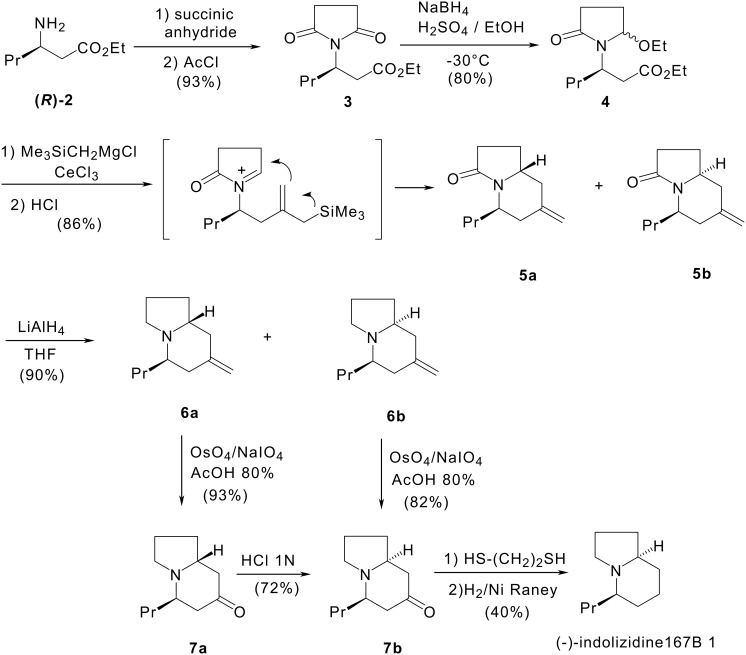
Enantioselective synthesis of (-)-indolizidine 167B by intramolecular allylsilane-*N*-acyliminium cyclisation.

The source of chirality was the aminoester **(*R*)-2** which was prepared according to Davies'methodology. [17] Synthesis of the indolizidine skeleton was carried out as shown in Scheme 2. Reaction of **(*R*)-2** with succinic anhydride and then with acetyl chloride in refluxing toluene gave imide **3**, then, **3** was reduced into ethoxylactam **4**. In the next step, **4** was treated with two equivalents of the cerium reagent derived from trimethylsilymethylmagnesium chloride and CeCl_3_. The mixture was then hydrolysed with 1N HCl to give methylenindolizidinones **5a** and **5b** in a 4:1 ratio. Reduction of the mixture of lactams **5a** and **5b** with lithium aluminium hydride gave methylenindolizidines **6a** and **6b** which were separated by flash chromatography.

Osmium tetroxide catalysed periodate oxidation of the olefinic bond of **6a** and **6b** led respectively to indolizidin-3-ones **7a** and **7b**. Upon treating an aqueous solution of **7a** with 1N HCl the thermodynamically more stable indolizidinone **7b** was obtained through a retro-Mannich fragmentation-cyclisation process. The last two steps were the conversion of **7b** into its dithiolane and subsequent desulfurisation using Raney nickel. The synthesis of (-)-indolizidine 167B **1** has been achieved in 7 steps with a 17% overall yield from ethyl (3*R*)-3-aminohexanoate **2** with an enantiomeric excess of 93%. [19]

#### I 1.2 Intermolecular cyclisation

**Scheme 3:**
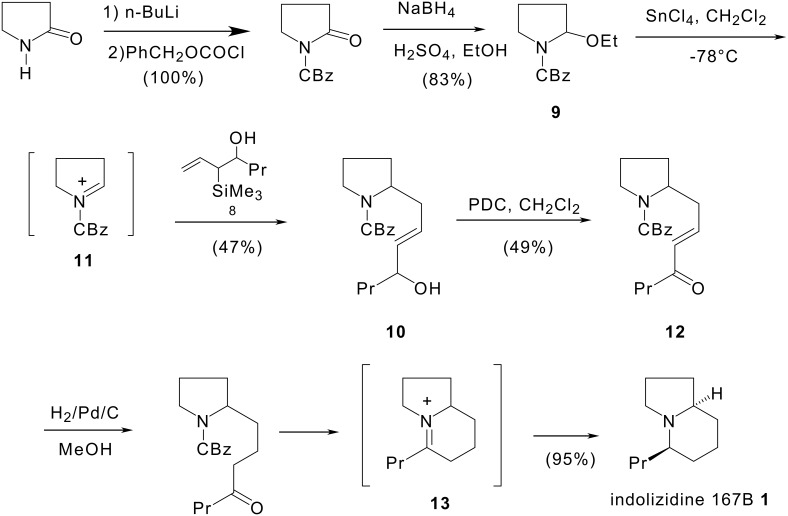
Synthesis of (±)-indolizidine 167B by intermolecular cyclisation of allylsilane-*N*-acyliminium cyclisation.

The intermolecular reaction between hydroxyalkyl-substituted allylsilanes and the acyliminium ion coming from pyrrolidin-2-one constitutes a new route to 5-substituted indolizidines (Scheme 3).

Hydroxyallylsilane **8** was synthesised as described [18] by reaction of the reagent prepared from allyltrimethylsilane, *sec*-butyllithium and titanium tetraisopropoxide with aldehydes. The key step of the synthesis is the intermolecular addition of the allylsilyl functional group of alcohol **8** on the acyliminium ion derived from ethoxycarbamate **9**.

Treatment of a mixture of ethoxycarbamate **9** and hydroxyallylsilane **8** with one equivalent of stannic chloride resulted in the formation of **10** via the acyliminium ion intermediate **11**. Subsequent oxidation of alcohol **10** with pyridinium dichromate, then catalytic hydrogenation (H_2_ over Pd/C) of ketone **12** induced hydrogenolysis of the CBz group, reduction of the double bond of the side chain and reduction of the iminium ion intermediate **13** to give the indolizidine 167B **1.** [20]

The synthesis of (±)-indolizidine 167B has been achieved in five steps in 18% overall yield from pyrrolidin-2-one.

### I 2. 3,5-Disubstituted indolizidines

Most of the indolizidine alkaloids are disubstituted by alkyl chains at the 3,5 positions. These compounds have been attractive targets for synthesis because of their potential biological activities. [7] Accordingly, novel strategies for the preparation of substituted indolizidines have received considerable attention. [21–27]

The allylsilyl functional group is a weak carbon nucleophile for trapping *N*-acyliminium ions, thus providing a useful method for intramolecular carbon-carbon bond formation. [28–29] We have applied this methodology towards the synthesis of indolizidine alkaloids. (*vide supra*) We describe here a new approach to 3,5-disubstituted indolizidines based on an intermolecular addition of allylsilanes on an *N*-acyl iminium starting from *L*-pyroglutamic acid used as the chiral precursor.

Preparation of lactam **14** was accomplished starting from the commercially available *S*-(-)-pyroglutamic acid according to a previously described procedure. [30–31] Next, lactam **14** was protected (*n*-BuLi, benzyl chloroformate) then converted to ethoxycarbamate **15**, isolated as a mixture of two diastereomers according to Hiemstra's procedure. [32–33] Condensation of allylsilanes **16** onto iminium ion **A** generated *in situ* by treatment of **15** with stannous chloride led to compounds **17a** and **17b**. The next two steps were straightforward: oxidation (pyridinium dichromate) of **17a** and **17b** afforded α,β-ethylenic ketones **18a** and **18b**.

**Scheme 4:**
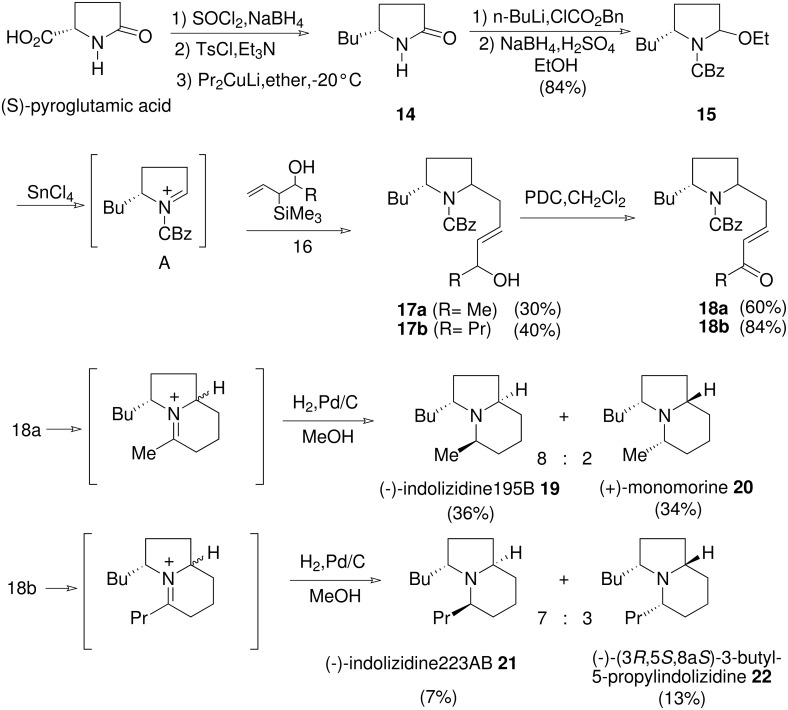
Synthesis of 3,5-disubstituted indolizidines from *L*-pyroglutamic acid.

On hydrogenation over palladium on carbon, **18a** gave a mixture of indolizidines **19** and **20** which were separated by flash chromatography. They were identified as (-)-indolizidine 195B and (+)-monomorine respectively. In the same manner, the hydrogenation of **18b** provided a mixture of isomers **21** and **22** respectively identified as (-)-indolizidine 223AB and (-)-(3*R*,5*S*,8a*S*)-3-butyl-5-propylindolizidine. [34] These four indolizidines were obtained in five steps with overall yields of about 8%.

### I 3 (-)-Dendroprimine

(-)-Dendroprimine **22** is an alkaloid isolated from *Dendrobium primulinum* Lindl (*Orchidaceae*) and shown to be a 5,7-dimethylindolizidine. [35] Its relative configuration was determined after the synthesis of the four racemic diastereomers of this indolizidine and a conformational analysis of these diastereomers has been discussed. [36–37] Its identification as (5*R*,7*S*,9*R*)-5,7dimethylindolizidine has been firmly established. [38] We describe here the first asymmetric synthesis of this alkaloid; [39] two other syntheses were recently published. [40–41]

**Scheme 5:**
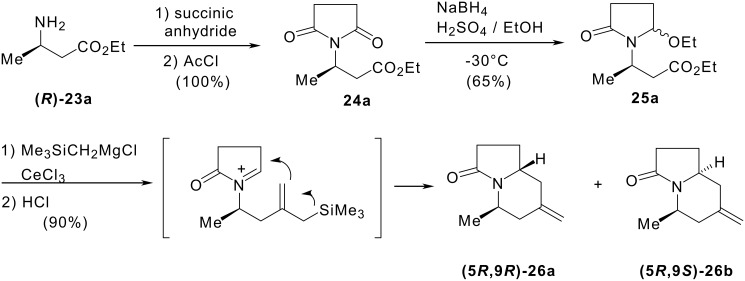
Access to indolizidine precursors of dendroprimine starting from chiral 2-aminopropanoate.

The first steps of our synthesis were carried out as shown in Scheme 5. The starting material was ethyl 2-aminopropanoate **23**. Chirality was introduced with isomers **(*R*)-23a** and **(*S*)-23b**, which were prepared by a Michael reaction according to Davies' procedure from ethyl crotonate and respectively (*R*)- and (*S*)-*N*-benzyl-α-methylbenzylamine. [17] Reaction of **23a** with succinic anhydride and then with acetyl chloride gave imide **24a**, it was then reduced into ethoxylactam **25a**. Compound **25a** was treated with the cerium reagent derived from trimethylsilylmagnesium chloride and cerium chloride. The mixture was then hydrolysed with 1N HCl to give methylenindolizidinones **26a** and **26b** in a 4:1 ratio.

**Scheme 6:**
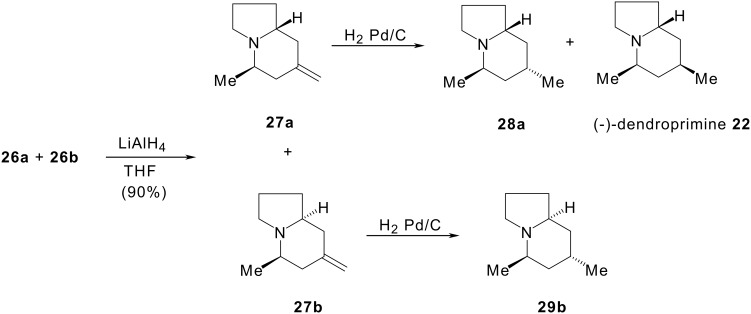
Access to (-)-dendroprimine by reduction with LiAlH_4_ of indolizidinones 26.

These diastereomers could not be separated. According to Scheme 6, in the first step the reduction of the lactam functional group of cyclisation products **26a** and **26b** with lithium aluminium hydride afforded a 4:1 mixture of methylenindolizidines **27a** and **27b** in quantitative yield. These isomers were separated. Palladium-catalysed hydrogenation of **27a** was found to be stereoselective, giving a mixture of **28a** and (-)-(dendroprimine) **22** in a 3:1 ratio. Using similar conditions, **27b** led to compound **29b**.

**Scheme 7:**
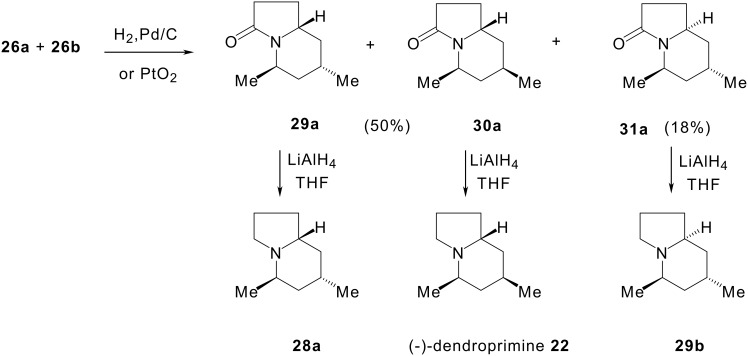
Access to (-)-dendroprimine by catalytic hydrogenation of indolizidinones 26.

Another way (cf. Scheme 7) was studied to access (-)-dendroprimine **22**: hydrogenation of the crude mixture of cyclisation products **26a** and **26b** over palladium on carbon provided a mixture of lactams **29a**, **30a** and **31a** in which isomer **30a** was preponderant (**29a**/**30a**/**31a** = 15:65:20). Flash column chromatography gave pure **31a** in 18% yield, but **29a** and **30a** could not be separated (50% yield). A mixture of the three isomers was used without purification for the next step. This mixture was then reduced with lithium aluminium hydride to give the indolizidines **28a**, **22** and **29b.** In conclusion, (-)-dendroprimine was obtained in five steps with overall yields of 17 and 20%.

## II Quinolizidines

### II 1-Myrtine and epimyrtine

(+)-Myrtine and (-)-epimyrtine are quinolizidine alkaloids isolated from *Vaccinium myrtillus* (Ericaceae). [4–5] Several syntheses of these compounds as racemic mixtures have been described, [5,42–44] but only three enantioselective syntheses of (+)-myrtine [43,45] and three syntheses of (-)-epimyrtine have been published. [46–47]

#### II 1.1 Synthesis of (±)-myrtine and (±)-epimyrtine

**Scheme 8:**
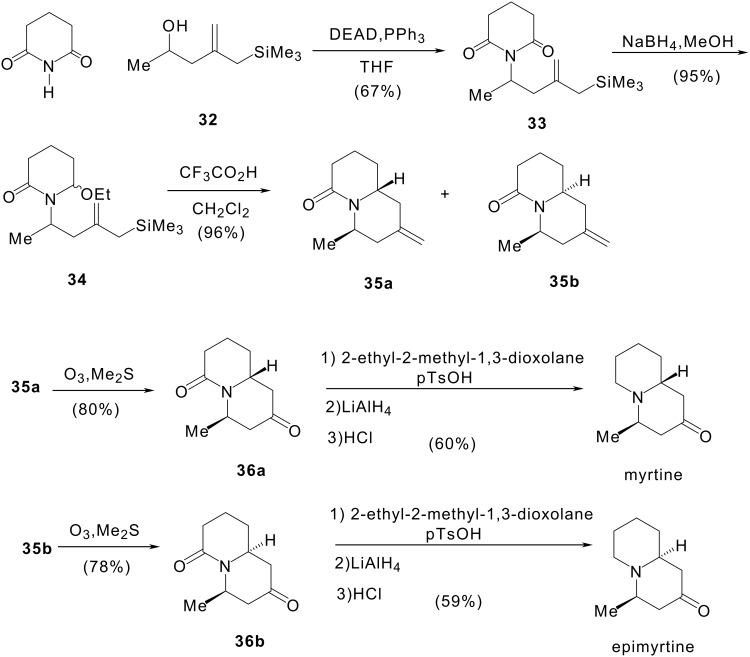
Synthesis of (±)-myrtine and (±)-epimyrtine.

These compounds have been prepared according to Scheme 8, the synthesis of hydroxyalkylallylsilane **32** is accomplished in 40% yield following Trost's procedure. [48] Reaction of glutarimide with alcohol **32** under Mitsunobu reaction conditions afforded imide **33** in 67% yield. Reduction of **33** was carried out with an excess of sodium borohydride in methanol at 0°C to give **34** as a mixture of two diastereomers which were not separated. The hydroxylactam **34** was then cyclised to the quinolizidine isomers **35a** and **35b** on treatment with 4 equiv. of trifluoroacetic acid in a 7:3 ratio. Then, ozonolysis of **35a** and **35b** followed by reduction with dimethylsulfide furnished respectively **36a** and **36b**. Protection of the carbonyl group by ketalisation with 2-ethyl-2-methyl-1,3-dioxolane and *p*-toluenesulfonic acid, reduction of the amide function with lithium aluminium hydride then quantitative removal of the protecting group (HCl treatment) afforded (±)-myrtine and (±)-epimyrtine. These syntheses were achieved in seven steps and 20% overall yield. [49]

#### II 1.2 Synthesis of (+)-myrtine and (-)-epimyrtine

**Scheme 9:**
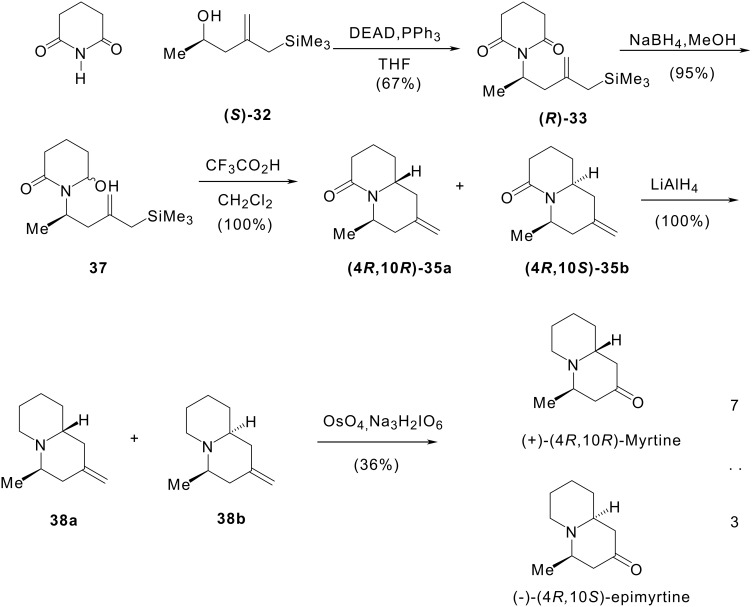
Enantioselective synthesis of (+)-myrtine and (-)-epimyrtine.

We used a similar strategy to prepare the enantiopure compounds starting from (*S*)-2-(hydroxypropyl)allyltrimethylsilane **32** (cf. Scheme 9). Compound **32** was obtained in quantitative yield by cerium mediated trimethylsilylmethylmagnesium chloride addition to ethyl (*S*)-3-hydroxybutanoate as we described. [50] The first three steps of the enantioselective synthesis were those previously described for the synthesis of racemic compounds (*vide supra*). Condensation of alcohol **32** with glutarimide under Mitsunobu conditions led to (+)-imide **(*R*)-33** in 67% yield. Reduction of **(*R*)-33** with sodium borohydride afforded hydroxylactam **37** as a 1:1 mixture of isomers in 95% yield. Treatment of hydroxylactam **37** with trifluoroacetic acid in methylene chloride gave a 7:3 mixture of the two isomeric bicyclic compounds **(4*R*,10*R*)-35a** and **(4*R*,10*S*)-35b** in quantitative yield. Reduction of this mixture of lactams with lithium aluminium hydride gave a 7:3 mixture of methylenquinolizidines **38a** and **38b** in quantitative yield. Osmium tetroxide-catalysed periodate oxidation of the olefinic bond of quinolizidines **38a** and **38b** under carefully controlled conditions led to a 7:3 mixture of the two diastereomeric alkaloids (+)-myrtine and (-)-epimyrtine. These alkaloids were obtained in five steps from (*S*)-2-(2-hydroxypropyl)allylsilane **32** with an overall yield of 23% and a high enantiomeric purity. This synthesis constitutes the first total synthesis of naturally occurring (-)-epimyrtine and confirms the configuration 4*R*,10*S* which was assigned previously to this compound. [51]

### II 2. Lasubines

The Lythraceae alkaloids constitute a large family of natural products, most of which contain 4-arylquinolizidine substructures. Among them are the quinolizidine alkaloids lasubine I and lasubine II which have been isolated from *Lagerstroemia subscotata* Koehne. [3] Numerous racemic [44,52–54] and asymmetric total syntheses of these alkaloids have been described. [43,55–64]

#### II 2.1. Synthesis of (±)-lasubine I and (±)-lasubine II

**Scheme 10:**
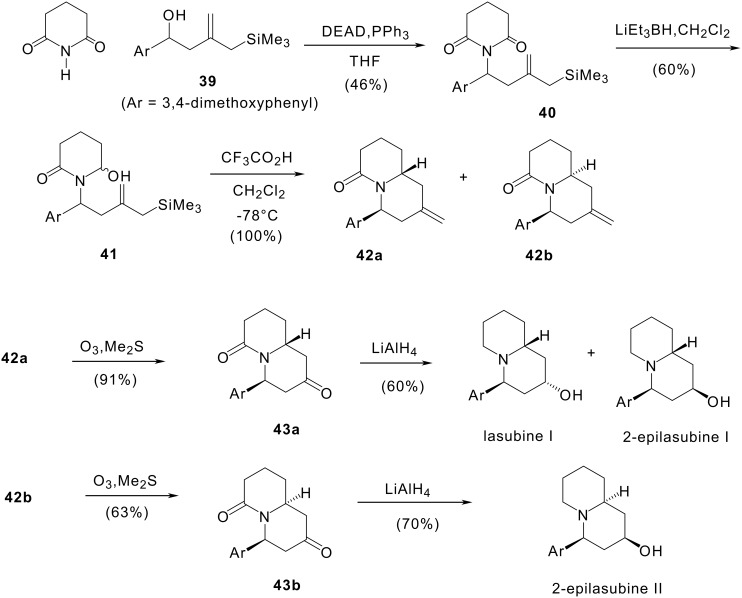
Synthesis of (±)-lasubines I and II and (±)-2-epilasubine II.

The first steps of our synthesis were carried out as shown in Scheme 10. The starting material was 2-(2-hydroxyethyl)allylsilane **39** which was prepared in 86% yield by indium mediated allylsilylation of 3,4-dimethoxybenzaldehyde, as already described. [65] Condensation of alcohol **39** with glutarimide under Mitsunobu conditions led to imide **40** in 46% yield. Reduction of **40** with diisobutylaluminium hydride afforded hydroxylactam **41** isolated as a mixture of isomers, a higher yield of a single isomer was obtained when using lithium triethylborohydride as reducing regent. The reduction had to be performed at -78°C to prevent formation of ring opening products. [66] Treatment of hydroxylactam **41** with trifluoroacetic acid in methylene chloride gave a mixture of isomeric bicyclic compounds **42a** and **42b** in a quantitative yield and a 4:1 ratio when the reaction was performed at -78°C.

Then, we examined two routes to the quinolizidine alkaloids lasubine I and lasubine II from methylenquinolizidinones **42a** and **42b**. They involved oxidation of the methylene group into a carbonyl which was then stereoselectively reduced to the hydroxyl group. The shortest route consisted of the ozonolysis of the methylene group followed by the simultaneous reduction of the two carbonyl groups of keto lactams **43a** and **43b**. Thus, treatment of **42a** with ozone then with dimethyl sulfide afforded the expected keto lactam **43a** in 91% yield. Ozonolysis of **42b** led to keto lactam **43b** in 63% yield. Reduction of **43a** with lithium aluminium hydride afforded in 60% yield a 1:1.2 mixture of lasubine I and 2-epilasubine I which were separated as their acetates. In the same way, reduction of **43b** gave 2-epilasubine II in 70% yield.

**Scheme 11:**
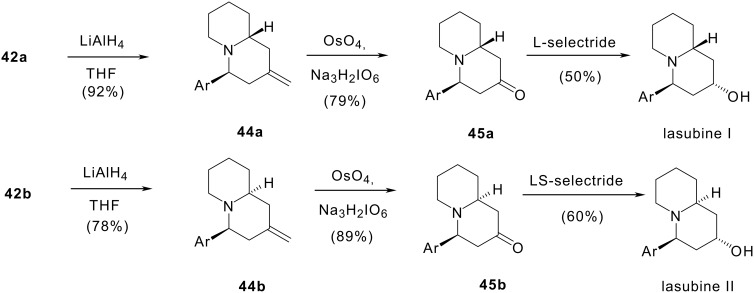
Synthesis of (±)-lasubine I and II.

In order to circumvent the stereochemical difficulty we decided to reduce first the lactam group (cf. Scheme 11) to obtain quinolizidines whose conformation should not be distorted by the junction with the piperidone ring. Lactams **42a** and **42b** were reduced with lithium aluminium hydride to give methylenquinolizidines **44a** and **44b** in 92% and 78% yields respectively. Osmium tetroxide catalysed periodate oxidation of the olefinic bond of methylenquinolizidines **44a** and **44b** under carefully controlled conditions led to the already described 2-oxoquinolizidines **45a** and **45b** in 79% and 89% yields respectively. The final step is a reduction of the carbonyl group. The use of borohydride in the reduction of **45a** has been described to give lasubine in an excellent yield. [67–68] In our hands, this reaction afforded a 1:1 mixture of (±)-lasubine I and (±)-epilasubine I. Stereoselective reduction of quinolizidin-2-one **45a** to (±)-lasubine I was achieved in 50% yield with lithium tri-*sec*-butylborohydride (L-selectride). Quinolizidinone **45b** was selectively converted to (±)-lasubine II with lithium trisamylborohydride (LS-selectride) in 60% yield.

In conclusion, (±) lasubine I and (±)-lasubine II were obtained in six steps from 2-(2-hydroxyethyl)allylsilane **39** in 8% and 7.4% yields respectively.

#### II.2.2 Synthesis of (-)-lasubine I, (-)-lasubine II and (+)-subcosine II

**Scheme 12:**
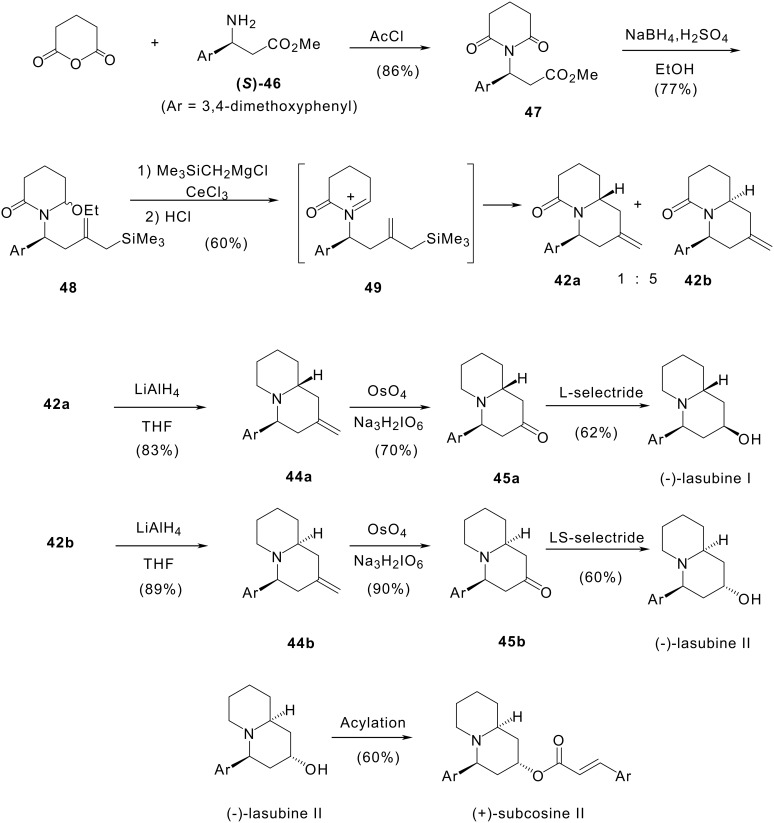
Enantioselective synthesis of (-)-lasubines I and II and (+)-subcosine.

A similar strategy was attempted from (+)-(3*R*)-ethyl 3-hydroxy-3-(3,4-dimethoxyphenyl)propionate but racemisation was observed during the Mitsunobu reaction. [69] So we developed another strategy to prepare these natural optically active compounds based on the intramolecular acyliminium ion allylsilane cyclisation of intermediate **49** generated from ethoxylactam **48**. Chirality is introduced with the β-aminoester **46**.

(*S*)-β-Aminoester **46** was prepared according to Davies'procedure. [17] Reaction of **46** with glutaric anhydride then with acetyl chloride in refluxing toluene gave imide **47** in 86% yield. Imide **47** was reduced into ethoxylactam **48** which was isolated as a mixture of two diastereomers. In the next step, ethoxylactam **48** was treated with the cerium reagent derived from CeCl_3_ and trimethylsilylmethylmagnesium chloride. The mixture was then hydrolysed with 1N HCl to give methylenquinolizidinones **42a** and **42b** in a 1:5 ratio and 60% yield. Reduction of lactams **42a** and **42b** with lithium aluminium hydride in refluxing THF for 12 h gave methylenquinolizidines **44a** and **44b** in 83% and 92% yields respectively. Osmium tetroxide catalysed periodate oxidation of the olefinic bond of **44a** and **44b** under carefully controlled conditions led to quinolizidin-2-ones **45a** and **45b** in 70 and 90% yields. The final step is a reduction of the carbonyl group. Stereoselective reduction of **45a** with L-selectride provided (-)-lasubine I in 62% yield. Quinolizidin-2-one **45b** was selectively converted to (-)-lasubine II with LS-selectride in 65% yield. Acylation of (-)-lasubine II with 3,4-dimethoxycinnamic anhydride gave (+)-subcosine II in 60% yield.(Scheme 12)

In conclusion, we have described the total synthesis of (-)-lasubine I, (-)-lasubine II and (+)-subcosine II using intramolecular cyclisation of *N*-acyliminium ion **(*S*)-49**. (-)-Lasubine I and (-)-lasubine II were obtained in six steps with overall yields of 7 and 14% respectively. (+)-Subcosine was prepared in seven steps with an overall yield of 9%. These three compounds were obtained with high enantiomeric purity. These results constitute the first total synthesis of naturally occurring (-)-lasubine II and (+)-subcosine II and unambiguously establish their absolute configuration as 2*S*,4*S*,10*S*.
